# Comparative metagenomic and untargeted metabolomic analyses reveal gut microbiota and metabolite alterations associated with diarrhea in neonatal Holstein calves in Anhui, China

**DOI:** 10.14202/vetworld.2026.2763-2776

**Published:** 2026-07-06

**Authors:** Xing Zhang, Hui Liu, Chaoyue He, Huiyang Chen, Huilin Zhang, Falei Li

**Affiliations:** 1 Rural Revitalization Collaborative Technology Service Center of Anhui Province, School of Biological and Food Engineering, Fuyang Normal University, Fuyang, China.

**Keywords:** alpha-ketoglutaric acid, calf diarrhea, gut microbiota, metagenomics, metabolomics, neonatal Holstein calves, precision livestock management, Campylobacter jejuni

## Abstract

**Background and Aim:**

Neonatal calf diarrhea remains a major cause of morbidity, mortality, and economic losses in the dairy industry. Although alterations in gut microbial communities have been implicated in calf diarrhea, the interactions between intestinal microbiota and metabolites in neonatal Holstein calves remain incompletely understood. This study aimed to characterize gut microbiome and metabolomic alterations associated with diarrhea and to explore the relationships between differential microorganisms and metabolites in neonatal Holstein calves from Anhui, China.

**Materials and Methods:**

Fecal samples were collected from four diarrheic and four healthy female Holstein calves younger than 2 weeks of age. Shotgun metagenomic sequencing was performed using the DNBSEQ-T7 platform, and untargeted liquid chromatography–tandem mass spectrometry metabolomics was used to characterize fecal metabolites. Multivariate analyses, biomarker identification, pathway enrichment, and correlation analyses were conducted to investigate associations between microbial taxa and metabolites.

**Results:**

Distinct microbial and metabolic profiles were observed between healthy and diarrheic calves. Analysis of similarities confirmed significant differences in microbial composition between groups (R = 0.7917, p = 0.025). Forty microbial biomarkers were identified, with *Campylobacter jejuni*, *Campylobacter coli*, and *Bacillus cereus* showing increased abundance in diarrheic calves, whereas beneficial taxa such as *Faecalibacterium* *prausnitzii* were enriched in healthy calves. Metabolomic analysis identified 377 differential metabolites, including 130 upregulated and 247 downregulated compounds in diarrheic calves. Pathway analysis indicated that D-glutamine and D-glutamate metabolism was the most affected pathway, together with alanine, aspartate, and glutamate metabolism, thiamine metabolism, taurine and hypotaurine metabolism, and cysteine and methionine metabolism. Five key metabolites, glutamate, α-ketoglutaric acid, thiamine, 3-sulfinoalanine, and S-adenosyl-L-homocysteine, were strongly associated with differentially abundant microorganisms. Correlation analyses demonstrated significant microbe–metabolite interactions, suggesting that dysbiosis contributes to metabolic disturbances during diarrhea.

**Conclusion:**

Neonatal calf diarrhea was associated with pronounced alterations in gut microbial communities and fecal metabolites. The enrichment of opportunistic pathogens and disruption of amino acid-related metabolic pathways highlight potential biomarkers and mechanistic links underlying gut dysbiosis. These findings provide novel insights into microbiota–metabolite interactions and may facilitate the development of targeted strategies to improve calf health and reduce economic losses in dairy production.

## INTRODUCTION

The incidence of calf diarrhea is notably high, and despite preventive measures implemented on most farms, it remains a leading cause of pre-weaning calf mortality and a major source of economic losses in the dairy industry [1, 2]. The global incidence of calf diarrhea ranges from 20% to 100%, with some farms reporting rates of 90%–100% [3]. According to the National Animal Health Monitoring System in the United States, 39% of calf deaths are attributed to diarrhea, whereas in South Korea, this figure rises to 53.4% [4, 5]. The causes of calf diarrhea are diverse, with infectious agents, including viruses, bacteria, and parasites, representing the primary etiologic factors [6].

The gut microbiota plays a crucial role in host health, contributing to essential physiological functions such as metabolism and immune regulation [7, 8]. Various metabolites produced by the gut microbiota, including short-chain fatty acids (SCFAs), provide nutritional support for host metabolism [9]. The gut microbiota also actively participates in the development of immune organs and the regulation of immune responses [10]. Furthermore, the gut microbiota contributes to the formation of the intestinal mucosal barrier, which is essential for resisting pathogen invasion and maintaining intestinal homeostasis [11, 12]. A healthy gut microbiota is indispensable for maintaining host immune homeostasis, whereas pathogenic infections can disrupt microbial composition, impair host balance, and increase disease susceptibility [13]. Gut symbionts contribute to intestinal microecological homeostasis by producing active metabolites, including SCFAs, antimicrobial peptides, and polymyxins, which inhibit the proliferation of opportunistic and exogenous pathogens [14]. In addition, these symbiotic microorganisms enhance innate immune responses to parasitic infections by promoting epithelial cell integrity and stimulating the production of both pro-inflammatory and anti-inflammatory mediators [15, 16].

During early life, the gut microbiota plays a pivotal role in intestinal development and immune maturation in calves. However, the gut microbiota of neonatal calves is immature and highly susceptible to pathogen invasion, thereby predisposing animals to diarrhea. During diarrhea, the gastrointestinal tract shifts from obligate anaerobic to facultative anaerobic bacteria, resulting in microbial dysbiosis [17]. Diarrheic calves commonly exhibit reduced microbial diversity and richness, accompanied by increased abundances of facultative anaerobes such as *Clostridium* spp. and *Escherichia coli* compared with healthy calves [4, 18]. *Cryptosporidium* spp. are among the major pathogens responsible for calf diarrhea and mortality [19]. Infection with *Cryptosporidium parvum* has been reported to alter the gut microbiota and significantly increase the abundance of *Clostridium* spp., with this abundance positively correlated with the severity of cryptosporidial infection [20]. Moreover, fluctuations in the abundance of Enterobacteriaceae are significantly associated with diarrhea, whereas the abundances of Firmicutes and Bacteroidetes are higher in healthy calves. In contrast, Proteobacteria are generally less abundant in healthy calves than in diarrheic calves [1, 21, 22].

It is noteworthy that pathogens capable of causing diarrhea can also be detected in healthy calves, indicating that their presence alone is not always indicative of disease [23]. In most cases, calf diarrhea is multifactorial, with multiple intestinal pathogens contributing to disease progression. Furthermore, therapeutic interventions may not restore gut microbial diversity in the short term [24]. Pathogenic bacteria can exploit microbe-derived carbon and nitrogen sources as nutrients and regulatory signals, thereby enhancing their own growth and virulence. This process induces intestinal inflammation, disrupts the gut environment, and further exacerbates dysbiosis [25, 26]. In turn, dysbiosis promotes the production of metabolites that favor the proliferation of pathogenic microorganisms. For example, *Salmonella enterica* and *E. coli* benefit from metabolites such as ethanolamine, lactate, glucuronate/galacturonate, propylene glycol, succinate, and L-serine generated during dysbiosis [27–30]. Previous metabolomic studies have demonstrated that intestinal inflammation alters amino acid metabolism. In calves, fecal concentrations of amino acids, including serine, alanine, valine, isoleucine, glycine, and leucine, as well as the expression of genes associated with vitamin and carbohydrate metabolism, are reduced during diarrhea [1]. Therefore, a comprehensive understanding of the interactions between the gut metagenome, metabolome, and host environment is essential for elucidating the mechanisms underlying the onset and progression of calf diarrhea [25].

Although previous studies have demonstrated that calf diarrhea is associated with alterations in intestinal microbial communities and metabolic profiles, most investigations have examined microbiota composition or metabolite changes independently. Consequently, the interactions between intestinal microorganisms and metabolites during neonatal calf diarrhea remain incompletely understood. In addition, integrated multi-omics studies that combine shotgun metagenomics and untargeted metabolomics remain limited, particularly in neonatal Holstein calves. Data from Anhui Province, an important dairy-producing region in eastern China, are scarce, and region-specific information regarding the relationships between microbial dysbiosis and metabolic disturbances is lacking. Furthermore, the mechanisms linking opportunistic pathogens to disruptions in amino acid-related metabolic pathways remain poorly elucidated. Therefore, comprehensive analyses integrating microbial and metabolomic datasets are required to identify potential biomarkers and provide mechanistic insights into host–microbe–metabolite interactions associated with neonatal calf diarrhea.

Therefore, the present study aimed to comprehensively characterize alterations in the gut microbiota and fecal metabolome associated with diarrhea in neonatal Holstein calves from Anhui Province, China, using shotgun metagenomic sequencing and untargeted metabolomics. In addition, the study aimed to investigate the relationships between differentially abundant microorganisms and metabolites, identify key metabolic pathways affected by diarrhea, and elucidate host–microbe–metabolite interactions associated with intestinal dysbiosis. These findings may facilitate the identification of potential biomarkers and provide a scientific basis for developing targeted strategies to improve calf health and reduce economic losses in dairy production.

## MATERIALS AND METHODS

### Ethical approval

The study protocol was reviewed and approved by the Research Ethics Committee of Fuyang Normal University, Fuyang, China, on December 1, 2023 (Approval No. FYNU2023AP024). Before sample collection, written informed consent was obtained from the farm owners. The study involved only rectal fecal sample collection from neonatal Holstein calves and did not include invasive procedures, experimental infection, treatment administration, or euthanasia. All calves were maintained under routine farm management conditions, and sampling was performed by trained personnel using sterile procedures to minimize stress, discomfort, and risk of injury. Animal handling, sample collection, transportation, and storage were conducted in accordance with institutional animal welfare guidelines and accepted veterinary practices.

### Study period and location

The study was conducted in Anhui Province, China, a major dairy-producing region in eastern China. The calves included in the study were born between December 2023 and January 2024. Fecal sampling was performed on January 25, 2024. Following collection, the samples were transported on dry ice to the laboratory and stored at −80°C until further analyses.

### Study design

A comparative study was conducted to investigate alterations in the gut microbiota and fecal metabolome associated with neonatal calf diarrhea. Eight female Holstein calves younger than 2 weeks of age, maintained under identical management conditions, were enrolled in the study. Four calves with watery diarrhea constituted the diarrheic group (D1, D2, D3, and D4), whereas four clinically healthy calves with normal feces constituted the healthy group (H1, H2, H3, and H4). All calves were housed individually in straw-bedded pens (2.5 × 2 m) outside the barn, separated from their dams. None of the calves had been treated with vaccines, medications, or antibodies before sampling. Fecal samples were subsequently subjected to shotgun metagenomic sequencing and untargeted metabolomic analyses to characterize microbial and metabolic alterations associated with diarrhea.

### Experimental animals and sample collection

Fresh fecal samples were collected by rectal sampling from eight female Holstein calves reared on a dairy farm in Anhui Province, China. The study population consisted of four calves with watery diarrhea and four healthy calves with normal feces. Fresh fecal material was collected into individual sterile sealed bags labeled with the sampling date, location, and animal identification number. None of the calves had received vaccines, medications, or antibody treatments before sample collection.

Throughout the experimental period, all calves were maintained under identical environmental and management conditions and housed individually in straw-bedded pens (2.5 × 2 m) outside the barn, thereby preventing direct contact with their dams. On January 25, 2024, fecal samples were collected and transferred into 2 mL cryovials, transported on dry ice to the laboratory, and stored at −80°C until further processing.

### DNA isolation and metagenomic sequencing

High-quality genomic DNA was extracted from fecal samples using the QIAamp DNA extraction system (QIAGEN, Hilden, Germany). DNA quality was assessed by agarose gel electrophoresis to evaluate fragment size and degradation, NanoDrop One spectrophotometry (Thermo Fisher Scientific, Waltham, MA, USA) to determine purity, and a Qubit 4.0 fluorometer (Thermo Fisher Scientific) to measure DNA concentration.

DNA samples were fragmented to approximately 350 bp by sonication, followed by end polishing. Libraries were constructed using the VAHTS Universal Plus DNA Library Prep Kit for MGI (Catalog No. NDM617; Vazyme Biotech Co., Ltd., Nanjing, China). Library preparation included DNA fragmentation, end repair, A-tailing, adapter ligation, purification, and polymerase chain reaction amplification, with target insert sizes ranging from 250 to 300 bp. Initial library quantification was performed using the Qubit 4.0 fluorometer, and library quality was further assessed using an Agilent 2100 Bioanalyzer (Agilent Technologies, Santa Clara, CA, USA).

Qualified libraries were subjected to high-throughput sequencing on the DNBSEQ-T7 platform (Beijing Genomics Institute, Shenzhen, China), which uses reversible terminator-based sequencing-by-synthesis technology. Shotgun metagenomic sequencing was employed to achieve species-level taxonomic resolution and functional gene profiling beyond the capabilities of amplicon-based approaches commonly used in calf diarrhea studies. Fluorescence signals generated during sequencing were used to identify nucleotides, and raw sequence data were obtained for subsequent bioinformatic analyses.

### Metagenomic data analysis

Raw sequence reads were subjected to quality control using Trimmomatic software to obtain high-quality clean tags. Metagenomic assembly was performed using MEGAHIT software (https://github.com/voutcn/ megahit), and contigs longer than 300 bp were retained. Assembly quality was evaluated using QUAST (https://github.com/ablab/quast). Coding regions were predicted using MetaGeneMark software (version 3.26; https://exon.gatech.edu/GeneMark/), and redundant sequences were removed using Cluster Database at High Identity with Tolerance (CD-HIT) software (version 4.6.6; https://bio.tools/cd-hit) with a similarity threshold of 95% and a coverage threshold of 90%.

Nonredundant protein sequences were annotated against the Kyoto Encyclopedia of Genes and Genomes (KEGG), eggNOG (Evolutionary Genealogy of Genes: Non-supervised Orthologous Groups), Carbohydrate-Active enZYmes database, Comprehensive Antibiotic Resistance Database, and Virulence Factor Database using Basic Local Alignment Search Tool searches. Functional gene compositions among samples were compared, and principal component analysis (PCA) was performed using R software to visualize differences among samples. Samples located closer together in PCA plots were considered to possess more similar functional gene compositions.

Welch's *t*-test was used to assess intergroup differences, and principal coordinates analysis (PCoA) was conducted using four distance matrices derived from beta-diversity analyses. The cleaned reads were subsequently used for metagenomic assembly, gene-coding prediction, functional annotation, taxonomic classification, and abundance analyses.

Integration of untargeted liquid chromatography–tandem mass spectrometry (LC-MS/MS) metabolomics with KEGG and MetaboAnalyst pathway analyses enabled comprehensive detection of 2,523 metabolites and facilitated direct associations between microbial taxa and metabolic profiles.

### Untargeted metabolomics analysis of fecal samples

Approximately 25 ± 1 mg of each sample was mixed with 500 μL extraction solvent, vortexed for 30 s, homogenized for 4 min at 35 Hz, and sonicated for 5 min in a 4°C water bath. This process was repeated three times. Samples were then incubated at −40°C for 1 h to precipitate proteins and centrifuged at 14,000 × *g* for 15 min at 4°C. The resulting supernatants were collected for LC-MS/MS analysis.

Metabolomic analyses were performed using a Vanquish ultra-high-performance liquid chromatography system (Thermo Fisher Scientific) coupled with an Orbitrap Exploris 120 mass spectrometer (Thermo Fisher Scientific). The mobile phase consisted of water (pH = 9.75) containing ammonium acetate and ammonium hydroxide (25 mmol/L) and acetonitrile. The injection volume was 2 μL, and the autosampler temperature was maintained at 4°C.

The mass spectrometer was operated in information-dependent acquisition mode with the following parameters: sheath gas flow rate, 50 arbitrary units; auxiliary gas flow rate, 15 arbitrary units; capillary temperature, 320°C; full mass spectrometry resolution, 60,000; tandem mass spectrometry resolution, 15,000; and spray voltages of +3.8 kV and −3.4 kV in positive and negative ion modes, respectively.

Following preprocessing and denoising, metabolites were retained for further analyses. Missing values were replaced with one-half of the minimum value, and the data were normalized using internal standards. Peak number, sample identity, and normalized peak area data were imported into SIMCA 16.0.2 software (Sartorius Stedim Data Analytics AB, Umeå, Sweden) for multivariate analysis. Data were scaled and log-transformed to reduce noise and minimize the influence of highly variable metabolites.

PCA was used to visualize sample distributions, and the 95% confidence interval was used to identify potential outliers. Orthogonal partial least squares discriminant analysis (OPLS-DA) was employed to distinguish between groups and identify differential metabolites. Model robustness was evaluated by seven-fold cross-validation and 200 permutation tests. Variable importance in projection (VIP) values derived from the first principal component of OPLS-DA were used to evaluate variable contributions.

Metabolites with VIP values >1, p < 0.05, and fold changes >2 or <0.05 were considered significantly altered in diarrheic calves. Pathway enrichment analysis was performed using KEGG and MetaboAnalyst databases. Raw data were converted to mzXML format and processed using R scripts for peak detection, extraction, alignment, and integration. Metabolites were identified using the BiotreeDB database (version 3.0).

### Correlation analysis of gut microbial species and metabolites

Pearson correlation analysis was performed to investigate relationships among intestinal microorganisms across samples. The top 20 microbe–microbe correlations were extracted and visualized. In addition, Pearson correlation analysis was used to evaluate associations between intestinal microorganisms and metabolites, and the top 10 positive and negative microbe–metabolite correlations were selected for subsequent analyses.

## RESULTS

### Microbial shifts in feces of diarrheic calves

**Figure 1:**
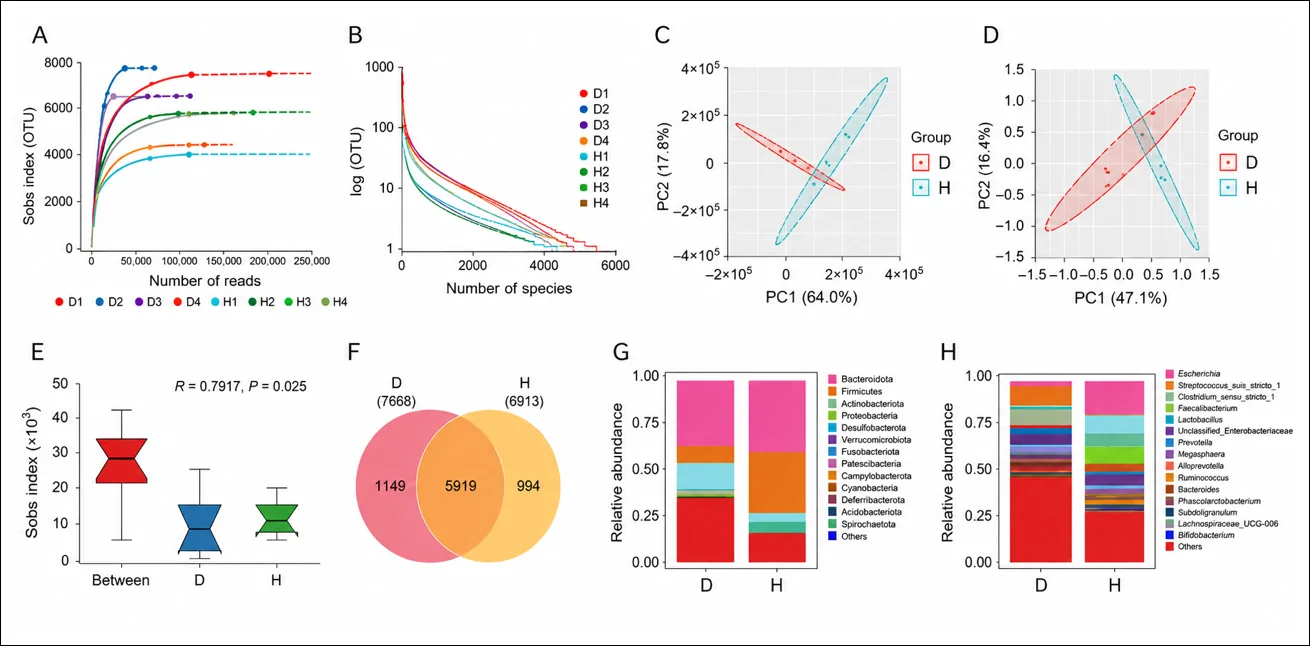
Microbial shifts in feces of diarrheic calves. (A) Dilution curve. (B) Abundance grade curve. (C) PCA plots for the H and D groups. (D) PCoA diagrams of the H and D groups. (E) Analysis of similarities test of differences in microbiome composition between the H and D groups. (F) Venn diagram showing the number of OTUs. (G) Bar chart of relative abundance at the phylum level for the H and D groups. (H) Bar chart of relative abundance at the genus level for the H and D groups.

The sequencing quality of all eight fecal samples was satisfactory (Table 1), and a total of 1,908,958 reads were obtained. The dilution curves flattened as sequencing depth increased, indicating adequate sequencing depth (Figure 1A). The abundance rank curve showed a steep decline, indicating a high proportion of dominant microbiota in calves' intestines (Figure 1B). PCA and PCoA plots revealed clustering of samples within the healthy (H) and diarrheic (D) groups, with clear separation between the two groups (Figure 1C and D). Intergroup analysis of similarities revealed a significant difference in microbial composition between the H and D groups (R = 0.7917, p = 0.025) (Figure 1E). Cluster analysis identified 8,062 operational taxonomic units (OTUs), with 7,068 OTUs in the D group and 6,913 OTUs in the H group (Figure 1F).

Compared with healthy calves, diarrheic calves showed marked changes in the intestinal microbiota at the phylum and genus levels (Figure 1G and H). At the phylum level, Firmicutes and Bacteroidetes were dominant in both the H and D groups, whereas Proteobacteria were more abundant in diarrheic calves (Figure 1G). At the genus level, *Staphylococcus*, *Streptococcus*, and *Klebsiella* were predominant in diarrheic calves, whereas *Prevotella*, *Faecalibacterium*, *Megasphaera*, and *Bacteroides* were predominant in healthy calves (Figure 1H).

### Differential microbial biomarkers and pathogen-associated taxa

**Figure 2:**
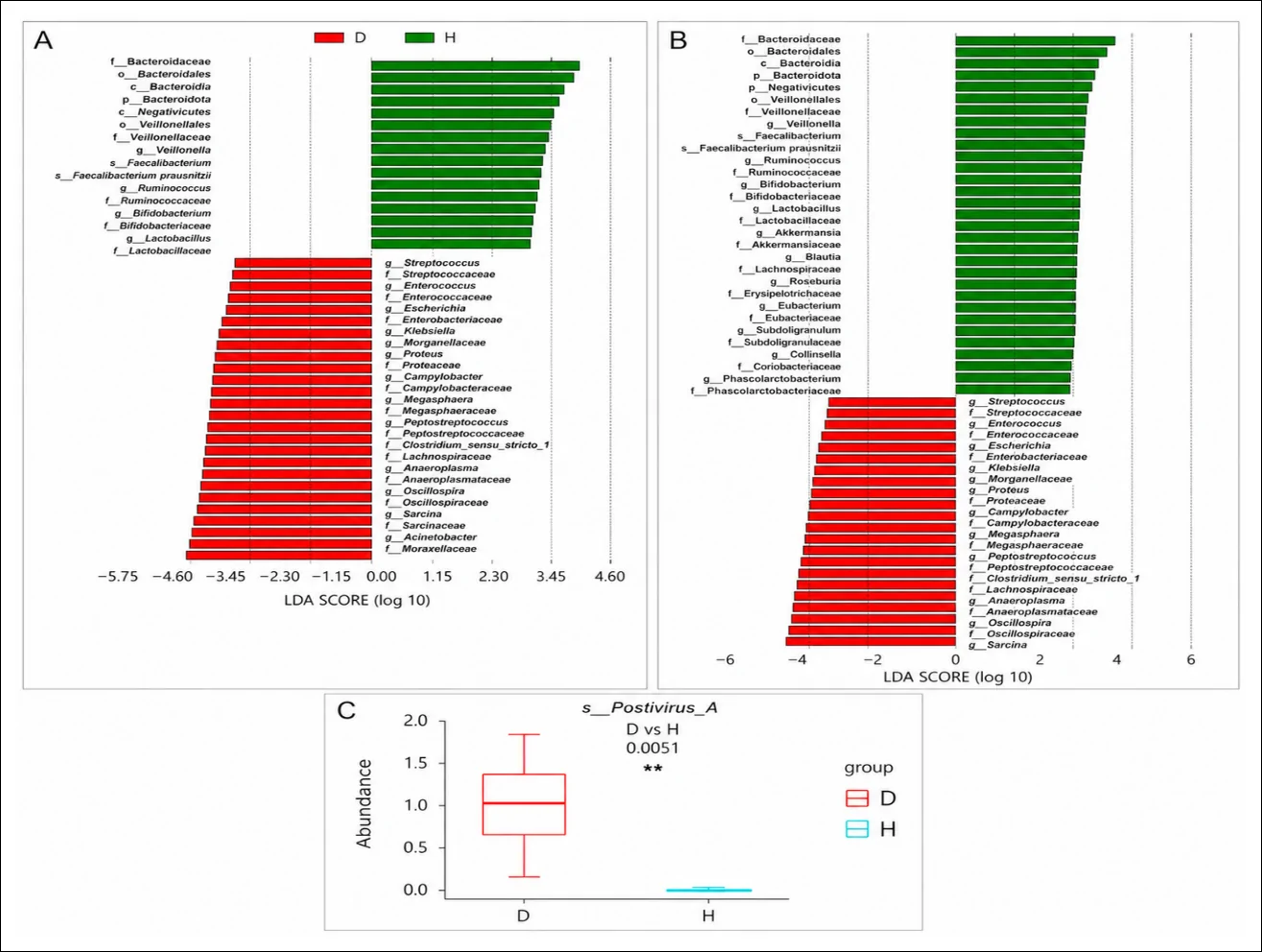
Analysis of differences in key intestinal microbes between diarrheic and healthy calves based on metagenomic data. (A) LEfSe analysis of the H and D groups, with LDA score >4 set as the criterion. (B) LEfSe analysis of the H and D groups, with LDA score >2 set as the criterion. (C) Relative abundance box plot of Pestivirus A analyzed using Metastats.

To identify microbial biomarkers associated with diarrhea, linear discriminant analysis effect size (LEfSe) analysis was performed between the two groups (Figure 2A). A total of 40 biomarkers were identified using a linear discriminant analysis (LDA) score >4 and p < 0.05. At the genus level, *Prevotella*, *Faecalibacterium*, and *Bifidobacterium* were significantly enriched in the H group, whereas *Staphylococcus*, *Streptococcus*, *Klebsiella*, and *Anaplasma* were significantly enriched in the D group. At the species level, *Faecalibacterium* *prausnitzii* was significantly enriched in the H group, whereas *Staphylococcus aureus*, *Streptococcus pneumoniae*, *Klebsiella pneumoniae*, *Clostridium botulinum*, and *Anaplasma phagocytophilum* were significantly enriched in the D group.

**Table 1:** Sample sequencing data.

**Group**	**Sample**	**Raw_reads**	**Raw_bases**	**Trimmed_reads**	**Trimmed_bases**	**Q20**	**Q30**	**GC**	**Effective**
D	D1	94509374	14176406100	91890106	13668699225	98.33%	94.77%	45.10%	96.42%
D	D2	102785308	15417796200	100845926	15037458553	98.83%	96.30%	43.30%	97.53%
D	D3	63546352	9531952800	61557426	9148114733	98.33%	94.94%	44.27%	95.97%
D	D4	100619046	15092856900	98797766	14726074331	98.84%	96.38%	44.15%	97.57%
H	H1	91008314	13651247100	89425790	13334611030	98.92%	96.53%	47.81%	97.68%
H	H2	92512342	13876851300	90473468	13465809980	98.49%	95.34%	50.83%	97.04%
H	H3	67411964	10111794600	65782912	9780048159	98.41%	95.14%	49.72%	96.72%
H	H4	115239436	17285915400	113006088	16846536985	98.78%	96.19%	46.91%	97.46%

No clear pathogenic species associated with diarrhea were detected in the D group when the LDA score threshold was set at >4. However, when the selection criterion was adjusted to an LDA score >2 and p < 0.05, potential diarrhea-associated species, including *Campylobacter jejuni*, *Campylobacter coli*, and *Bacillus cereus*, were identified (Figure 2B). Notably, even under the relaxed threshold of LDA >2, *C. jejuni*, *C. coli*, and *B. cereus* were enriched in diarrheic calves, whereas beneficial taxa such as *F. prausnitzii* were depleted. Metastats analysis revealed a significant difference in Pestivirus A abundance between the two groups (p < 0.01) (Figure 2C). High abundances of *Salmonella enterica*, *E. coli*, and *Clostridioides difficile*, which are known to cause calf diarrhea, were detected in both groups.

### Microbial correlation patterns

**Figure 3:**
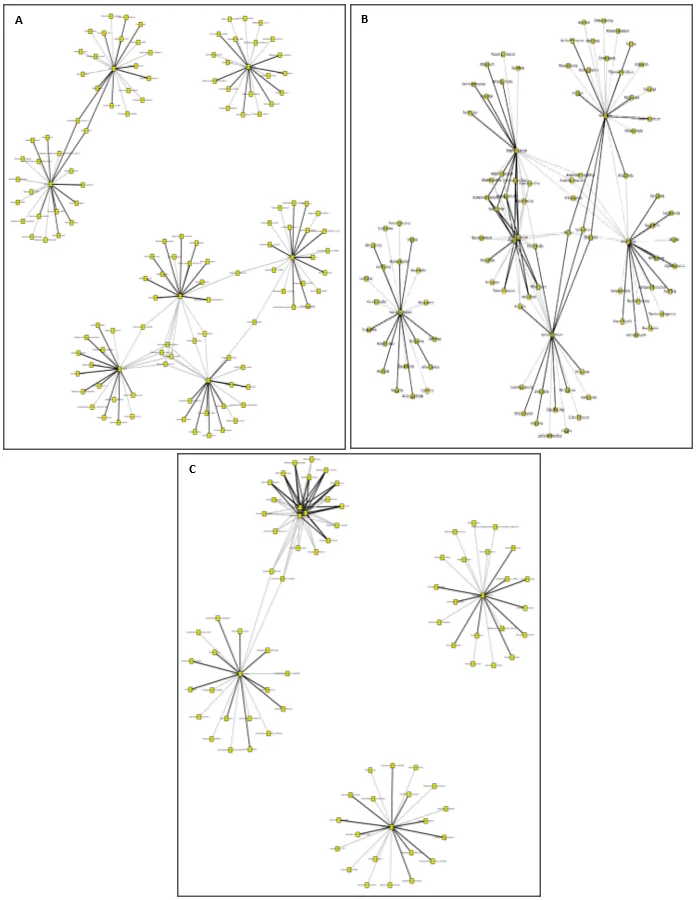
Microbial correlation analysis. (A) Network diagram of genera closely related to microorganisms with LDA score >4. (B) Network diagram of species closely related to microbial species with LDA score >4. (C) Network diagram of microbial species closely related to diarrheal pathogens. Yellow squares indicate microorganisms, and the color of the connecting lines indicates the correlation. Black indicates positive correlation, and gray indicates negative correlation.

*Anaerolactibacter* *massiliensis*, *Oribacterium* sp. oral taxon 078, *Pseudomonas* sp. SHC52, and *Mitsuokella* sp. AF21-1AC were significantly negatively correlated with *C. coli*, *C. jejuni*, and Pestivirus A. In addition, *Stenotrophomonas* sp. KAs_5-3 and *Shigella sonnei* showed significant positive correlations with *E. coli* and *S. enterica*, whereas *Olsenella* sp. AM30-3LB, *Ligilactobacillus salivarius*, *Bifidobacterium* sp. 56_9_plus, *Butyrivibrio* sp. NC3005, and *Acetanaerobacterium* *elongatum* showed significant negative correlations with *B. cereus* (Figure 3A).

Microbial correlation analysis showed that *Rothia*, *Syntrophus*, and *Olegusella* were significantly positively correlated with *Bifidobacterium* and *Prevotella*, whereas *Rothia* was significantly negatively correlated with *Anaplasma*, *Klebsiella*, and *Streptococcus* (Figure 3B). *S. pneumoniae*, *K. pneumoniae*, and *C. botulinum* were positively correlated with each other. These three species were negatively correlated with *Olsenella* spp. (*Olsenella* sp. TM06-36, *Olsenella* *scatoligenes*, and *Olsenella* sp. AM30-3LB), *Alistipes senegalensis*, *Lachnospiraceae bacterium* JC7, and *Anaerotruncus* sp. CAG:390, whereas they were positively correlated with *Millerozyma* *farinosa*, *Herbaspirillum* sp. VT-16-41, *Brachyspira* *hyodysenteriae*, and *Enterobacter mori* (Figure 3C). *S. pneumoniae*, *K. pneumoniae*, *C. botulinum*, and *A. phagocytophilum* showed significant positive correlations with *B. cereus*, *C. coli*, and Pestivirus A, whereas *F. prausnitzii* showed negative correlations with these three diarrheic pathogens.

### Metabolic shifts in feces of diarrheic calves

**Figure 4:**
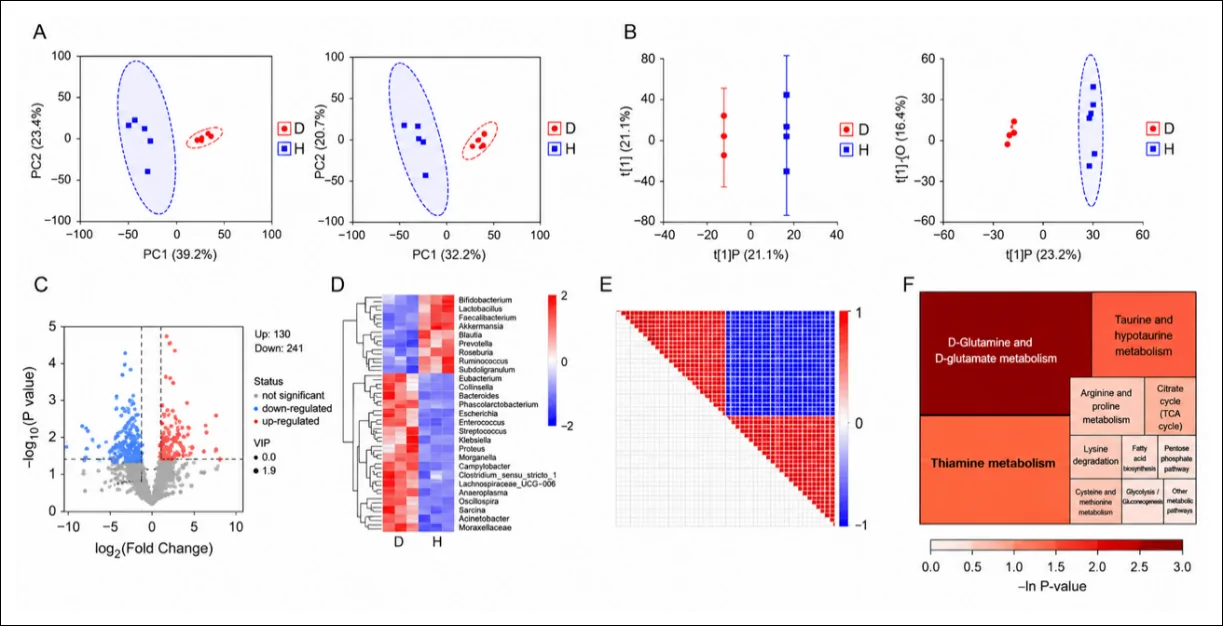
Metabolic shifts in fecal samples of diarrheic calves. (A) PCA analysis of fecal samples in the D and H groups under positive ion mode (left) and negative ion mode (right). (B) OPLS-DA scores of fecal samples in the D and H groups under positive ion mode (left) and negative ion mode (right). (C) Volcano plot depicting differentially expressed metabolites. The size of each point indicates the VIP value; blue points indicate downregulated differential metabolites, and red points indicate upregulated differential metabolites. (D) Heatmap of hierarchical clustering of differential metabolites. The x-axis indicates different experimental groups, and the y-axis indicates differential metabolites compared within each group. Red indicates high expression in the group, whereas blue indicates low expression. (E) Correlation between differentially expressed metabolites. In the correlation map, color indicates the correlation coefficient. Positive correlations are shown in red, and negative correlations are shown in blue. An asterisk was added to circles to indicate p > 0.05 for the correlation coefficient. (F) Rectangular tree analysis of metabolic pathways. Square size indicates the influence factor of the pathway in topological analysis; larger squares indicate greater influence. Square color indicates the p-value of the enrichment analysis after log transformation; darker color indicates smaller p-values and more significant enrichment.

A total of 2,523 metabolites were obtained after quality control assessment. The metabolic PCA models of fecal samples from diarrheic and healthy calves showed clear separation in both positive- and negative-ion modes, indicating that the models were stable and reliable (Figure 4A). OPLS-DA models established in both ion modes showed R2Y and Q2 values close to 1, indicating high predictive capability. These models also showed good robustness, with no evidence of overfitting (Figure 4B).

The threshold for selecting differential metabolites was set at p < 0.05, VIP > 1, and fold change > 2 or < 0.05. A total of 377 differential metabolites were identified between the D and H groups in both positive and negative ion modes, including 130 upregulated and 247 downregulated metabolites (Figure 4C). Cluster analysis of differential metabolites revealed that samples within the same group clustered closely (Figure 4D), indicating that the selected significant metabolites were appropriate for subsequent analysis. Correlation analysis of the top 50 differentially regulated metabolites between groups showed significant correlations (Figure 4E). For example, 1-O-hexadecyl-sn-glycero-3-phosphocholine was significantly positively correlated with falimint, annoglabasin A, and desmethyleneparoxetine, whereas it was significantly negatively correlated with 5-(2-thienylmethylene)-2,4,6(1H,3H,5H)-pyrimidinetrione and formamidopyrimidine nucleoside triphosphate.

Based on the pathway enrichment heatmap, pathways with higher enrichment levels were selected for further analysis (Figure 4F). Comprehensive analysis of differential metabolic pathways showed that calf diarrhea primarily affected D-glutamine and D-glutamate metabolism; alanine, aspartate, and glutamate metabolism; thiamine metabolism; taurine and hypotaurine metabolism; and cysteine and methionine metabolism. Five differential metabolites were enriched in these pathways: glutamate, alpha-ketoglutaric acid, thiamine, 3-sulfinoalanine, and S-adenosyl-L-homocysteine. Differential pathway analysis specifically highlighted D-glutamine and D-glutamate metabolism as the primary affected pathway, differing from previously reported purine or glycerophospholipid disruptions in other breeds or regions.

### Correlations between microbiota and metabolites in calf feces

**Figure 5:**
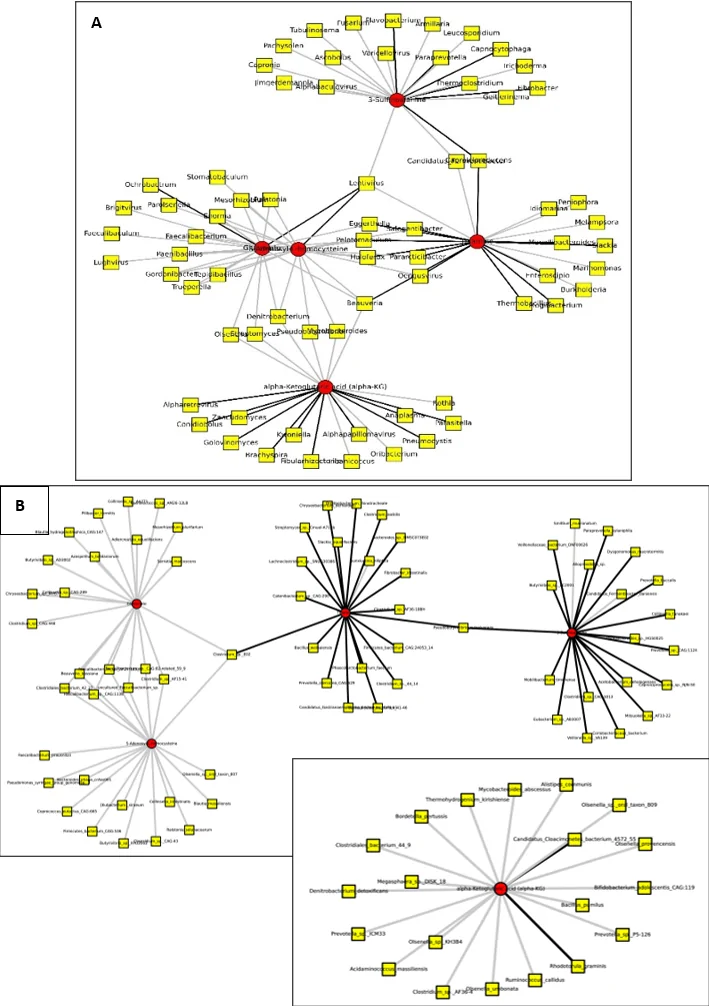
Correlations between microbiota and metabolites in fecal samples of healthy and diarrheic calves. (A) Bacterial genera with significantly different abundances that were significantly correlated with the core metabolites (p < 0.05). (B) Bacterial species with significantly different abundances that were significantly correlated with the core metabolites (p < 0.05). Yellow squares indicate microorganisms, red circles indicate core metabolites, and connecting line color indicates the correlation. Black indicates positive correlation, and gray indicates negative correlation.

To explore correlations between gut microbiota and metabolites in calf feces, microorganisms with significantly different abundances were analyzed in relation to five core metabolites: glutamate, alpha-ketoglutaric acid, thiamine, 3-sulfinoalanine, and S-adenosyl-L-homocysteine. Twenty microorganisms, including *Caproiciproducens*, *Candidatus fermentibacter*, and *Paraprevotella* spp., showed significant correlations with these five metabolites (p < 0.05) (Figure 5A). *Beauveria* and *Denitrobacterium* were significantly negatively correlated with glutamate, S-adenosyl-L-homocysteine, and alpha-ketoglutaric acid. *Lentivirus* showed significant positive correlations with glutamate and S-adenosyl-L-homocysteine. Both *Lentivirus* and *Candidatus fermentibacter* exhibited significant negative correlations with 3-sulfinoalanine and thiamine, whereas *Caproiciproducens* showed significant positive correlations with both metabolites. *Clostridium* sp. E02 was significantly negatively correlated with glutamate and S-adenosyl-L-homocysteine but positively correlated with thiamine. *Pseudobutyrivibrio* *xylanivorans* showed significant positive correlations with both 3-sulfinoalanine and thiamine (Figure 5B).

*A. phagocytophilum*, *C. coli*, *C. jejuni*, Pestivirus A, *S. pneumoniae*, *C. botulinum*, *K. pneumoniae*, and *B. cereus* showed significant negative correlations with 3-sulfinoalanine. *A. phagocytophilum*, *S. pneumoniae*, *C. botulinum*, *K. pneumoniae*, *C. coli*, and Pestivirus A were significantly positively correlated with alpha-ketoglutaric acid. *F. prausnitzii* was significantly negatively correlated with glutamate and S-adenosyl-L-homocysteine and significantly positively correlated with alpha-ketoglutaric acid. Integrated analysis revealed novel negative associations between beneficial taxa and diarrheagenic pathogens/metabolites, as well as specific links between pathogens and elevated alpha-ketoglutaric acid, providing new evidence for competitive exclusion mechanisms.

## DISCUSSION

### Gut microbial dysbiosis in diarrheic calves

The present study comprehensively characterized the gut microbiome and fecal metabolite profiles of diarrheic and healthy neonatal Holstein calves and analyzed associations between gut microbiota and metabolites in the context of calf diarrhea. This study provides original integrative multi-omics evidence from Anhui Province, China, showing that neonatal calf diarrhea is associated not only with the classical expansion of Proteobacteria but also with specific enrichment of *Campylobacter* spp. and *Bacillus* spp., together with glutamine/glutamate metabolic reprogramming. These findings complement and extend previous observations reported in other Chinese breeds and regions.

Firmicutes and Bacteroidetes are considered core members of the bovine microbiota and play crucial roles in cellulose digestion and in maintaining intestinal health. In the present study, Firmicutes and Bacteroidetes were predominant in both healthy and diarrheic calves. Proteobacteria are part of the intestinal microbiota and may participate in metabolism under normal conditions; however, increased abundance of this phylum may indicate underlying gut health disturbances [31]. Accordingly, Proteobacteria were more abundant in diarrheic calves.

In healthy calves, *Prevotella*, *Faecalibacterium*, and *Bifidobacterium* were significantly enriched. These core bacterial genera are essential to the bovine gut because they contribute to SCFA production, reduce intestinal inflammation, maintain microbial balance, and ultimately support digestive efficiency and productivity. Beneficial bacteria such as *Bifidobacterium* can inhibit pathogen growth, reduce intestinal permeability, maintain intestinal barrier integrity, and alleviate inflammation by increasing SCFA levels [32, 33]. Another beneficial species enriched in healthy calves was *F. prausnitzii*, which exerts anti-inflammatory effects by inhibiting nuclear factor-kappaB activation and interleukin-8 production via its metabolite, butyrate [34, 35].

Interestingly, healthy calves did not exhibit diarrheal symptoms despite showing high abundances of diarrhea-associated pathogens, including *C. jejuni*, *Salmonella enterica*, *C. coli*, Pestivirus A, *E. coli*, *B. cereus*, and *C. difficile*. This finding suggests that the presence of a pathogen alone may be insufficient to induce diarrhea and that microbial community structure may influence disease expression. The absence of clinical signs in healthy calves may be attributed to the enrichment of beneficial microorganisms exerting antagonistic effects against diarrheal pathogens. Consistent with this interpretation, correlation analysis showed that *F. prausnitzii* was negatively correlated with *B. cereus*, *C. coli*, and Pestivirus A.

The diarrheic group showed high abundances of previously reported pathogenic bacteria, including *Streptococcus pneumoniae* [36], *K. pneumoniae* [37, 38], *C. botulinum* [39], and *A. phagocytophilum* [40]. In addition, *C. jejuni*, *C. coli*, and *B. cereus* were identified as potential diarrhea-associated pathogens in calves with diarrhea. A notable observation from the correlation analysis was that *Alistipes* *putredinis* was negatively correlated with *A. phagocytophilum*, whereas *Alistipes senegalensis* was negatively correlated with *S. pneumoniae*, *K. pneumoniae*, and *C. botulinum*. Both *A. putredinis* and *A. senegalensis* possess anti-inflammatory effects in the gut and contribute to intestinal health and stability [41]. These findings further support the concept that beneficial species contribute to intestinal homeostasis and immunity by counteracting pathogenic bacteria. Overall, the gut microbiome of diarrheic calves appeared dysbiotic, characterized by a shift toward pathogenic microbes and significant changes in microbial structure and composition compared with that of healthy calves.

### Metabolic reprogramming associated with calf diarrhea

The interaction between the microbiota and host is mediated largely through microbial metabolites. Gut microbial dysbiosis alters the intestinal metabolic environment, thereby affecting host physiological processes and immune status. Inflammation-associated dysbiosis can change bacteria-derived metabolites in the gastrointestinal tract of calves and create an environment that favors the growth of pathogenic Enterobacteriaceae [1, 42]. In the present study, differential metabolite analysis identified five pathways and corresponding metabolites significantly affected by diarrhea: D-glutamine and D-glutamate metabolism (glutamate), alanine, aspartate, and glutamate metabolism (alpha-ketoglutaric acid), thiamine metabolism (thiamine), taurine and hypotaurine metabolism (3-sulfinoalanine), and cysteine and methionine metabolism (S-adenosyl-L-homocysteine).

Glutamate, alpha-ketoglutaric acid, and S-adenosyl-L-homocysteine were significantly increased in diarrheic calves, suggesting changes related to intestinal stress, tissue repair, and immune responses. In contrast, reduced levels of thiamine and 3-sulfinoalanine may indicate impaired intestinal metabolism during diarrhea. These metabolic alterations may serve as potential biomarkers for the diagnosis and management of diarrheic calves.

Correlation analysis between microorganisms and metabolites showed that several differentially abundant genera, including *Faecalibacterium*, *Gordonibacter*, *Pelotomaculum*, and *Enorma*, were negatively correlated with glutamate and S-adenosyl-L-homocysteine. *Faecalibacterium* [43] and *Gordonibacter* [44] have been associated with anti-inflammatory effects, whereas information on *Pelotomaculum* and *Enorma* remains limited. Further investigation of these correlations may improve understanding of microbiome–metabolome interactions and the mechanisms of gut dysbiosis in diarrheic calves.

### Practical and regional relevance

The integrated microbial and metabolomic patterns observed in this study provide region-specific evidence from Anhui Province, a key dairy-producing area in eastern China. The enrichment of *Campylobacter* spp. and *Bacillus* spp., combined with disruption of glutamine/glutamate, thiamine, taurine/hypotaurine, and sulfur amino acid pathways, suggests potential targets for early diagnosis and precision intervention. These findings may assist in developing biomarker-based monitoring strategies, improving calf health management, and reducing economic losses associated with neonatal calf diarrhea.

### Strengths and limitations

A major strength of this study is the use of an integrative multi-omics approach that combines shotgun metagenomic sequencing and untargeted metabolomics, enabling the simultaneous identification of microbial shifts, metabolic disturbances, and microbe–metabolite correlations. This approach provides broader mechanistic insight than studies relying only on microbial composition or metabolite profiling.

However, several limitations should be considered when interpreting the results. First, the sample size was small, and only a limited number of fecal samples were collected. Differences in diarrhea severity among calves may also have affected the results. Second, only rectal fecal samples were collected, and microbiota and metabolites from other intestinal regions were not examined. Therefore, the findings may not fully represent gut microbial and metabolic changes under different feeding and management conditions. Third, blood biochemical parameters were not assessed; therefore, calf health status was inferred only from the presence or absence of diarrhea. Healthy calves may have had stronger immune responses that prevented diarrhea despite exposure to pathogens. Finally, the study focused on very young calves in the early stage of pathogen exposure, which may have minimized individual variation in gut microbiota and metabolites and limited differences in microbial alpha diversity. Some healthy calves may also have been in an early preclinical stage of infection.

### Future perspectives

Future studies should include larger calf populations, multiple farms, and longitudinal sampling to validate the identified microbial and metabolic biomarkers. Additional analyses of blood parameters, host immune responses, intestinal tissue, and functional microbial activity would help clarify causal mechanisms. Experimental validation of key taxa and metabolites is also needed to determine whether they directly contribute to the pathogenesis of diarrhea or serve as indicators of disease-associated dysbiosis. Such studies may support the development of targeted nutritional, probiotic, or management-based interventions to prevent and control neonatal calf diarrhea.

## CONCLUSION

This study demonstrated that neonatal calf diarrhea is associated with pronounced alterations in both the gut microbiota and fecal metabolome. Diarrheic calves exhibited enrichment of opportunistic and pathogenic microorganisms, including *C. jejuni*, *C. coli*, and *B. cereus*, together with depletion of beneficial taxa such as *F. prausnitzii*. Metabolomic analysis identified 377 differential metabolites and revealed that D-glutamine and D-glutamate metabolism, alanine, aspartate, and glutamate metabolism, thiamine metabolism, taurine and hypotaurine metabolism, and cysteine and methionine metabolism were among the pathways most affected by diarrhea. Correlation analyses further demonstrated significant associations between specific microorganisms and metabolites, highlighting the importance of microbiome–metabolome interactions in the development of intestinal dysbiosis.

In conclusion, this study provides novel integrative multi-omics evidence that neonatal calf diarrhea in Holstein calves is characterized by microbial dysbiosis and metabolic reprogramming. The enrichment of pathogenic taxa and disruption of amino acid-related metabolic pathways provide mechanistic insights into disease development and identify promising biomarkers and intervention targets. These findings advance current understanding of host–microbe–metabolome interactions in neonatal calf diarrhea and provide a foundation for developing precision strategies to enhance calf health, productivity, and disease management in dairy systems.

## DATA AVAILABILITY

The datasets generated and analyzed during the present study have been deposited in the National Center for Biotechnology Information (NCBI) under BioProject accession number PRJNA1203373**.** The raw sequencing data are publicly available in the Sequence Read Archive (SRA) under accession numbers SRR31880828–SRR31880835.

## GENERATIVE AI DECLARATION

The authors declare that no generative artificial intelligence or AI-assisted technologies were used in the writing, analysis, or preparation of this manuscript.

## AUTHORS’ CONTRIBUTIONS

HZ and FL: Conceptualization, methodology, investigation, data curation, formal analysis, writing—original draft, and writing—review and editing. XZ and FL: Conceptualization, methodology, writing—original draft, and writing—review and editing. HL, CH, HC, and HZ: Formal analysis, data curation, writing—original draft, and writing—review and editing. All authors have read and approved the final version.
